# The relative contribution of shape and colour to object memory

**DOI:** 10.3758/s13421-020-01058-w

**Published:** 2020-06-15

**Authors:** Irene Reppa, Kate E. Williams, W. James Greville, Jo Saunders

**Affiliations:** 1 Wales Institute for Cognitive Neuroscience, Department of Psychology, Swansea University, Swansea, SA2 8PP UK; 2 Department of Psychology, University of Wales, Trinity St. David, UK; 3 Faculty of Life Sciences and Education, University of South Wales, Treforest, UK; 4 School of Psychological Sciences and Health, University of Strathclyde, Strathclyde, UK

**Keywords:** Object recognition, Retrieval-induced forgetting, Object colour, Object shape, Novel objects

## Abstract

The current studies examined the relative contribution of shape and colour in object representations in memory. A great deal of evidence points to the significance of shape in object recognition, with the role of colour being instrumental under certain circumstances. A key but yet unanswered question concerns the contribution of colour relative to shape in mediating retrieval of object representations from memory. Two experiments (N=80) used a new method to probe episodic memory for objects and revealed the relative contribution of colour and shape in recognition memory. Participants viewed pictures of objects from different categories, presented one at a time. During a practice phase, participants performed yes/no recognition with some of the studied objects and their distractors. Unpractised objects shared shape only (Rp–Shape), colour only (Rp–Colour), shape and colour (Rp–Both), or neither shape nor colour (Rp–Neither), with the practised objects. Interference effects in memory between practised and unpractised items were revealed in the forgetting of related unpractised items – *retrieval-induced forgetting*. Retrieval-induced forgetting was consistently significant for Rp–Shape and Rp–Colour objects. These findings provide converging evidence that colour is an automatically encoded object property, and present new evidence that both shape and colour act simultaneously and effectively to drive retrieval of objects from long-term memory.

## Introduction

Apart from its aesthetic value in visual perception, colour can play an important role in visual processing, such as helping segment objects from the background (e.g., flowers from foliage; see, e.g., Davidoff, 1991; Gegenfurtner & Rieger, 2000; Gegenfurtner, Wichmann, & Sharpe, 1998; Wichmann, Sharpe, & Gegenfurtner, 2002). Colour processing has practical consequences for survival, such as when it aids discrimination of ripe fruit among foliage (e.g., Mollon & Jordan, 1988; Regan, Julliot, Simmen, Vienot, Charles-Dominique, & Mollon, 2001). Yet, humans can lead successful lives even without full colour information as colour deficiency in its many forms demonstrates (e.g., Gegenfurtner et al., 1998).

A similarly ill-defined role for colour exists in object identification where it is not always found to make a contribution. Young children rely on shape, not colour or texture, in tasks requiring them to extend a novel count noun (e.g., ‘Dax’) from one object to another (e.g., Landau, Smith, & Jones, 1988). This shape bias is also found in adults (e.g., Landau et al., 1988) and in artificial neural networks (e.g., Ritter, Barrett, Santoro, & Botvinick, 2017). In adults, object naming, for example “What is this object?”, object categorisation, for example “Is this a fruit or an animal?” or object verification tasks, for example “Is this object a lemon?”, is highly dependent on object shape (e.g., Biederman & Ju, 1988; Rosch, Mervis, Gray, Johnson, & Boyes-Braem, 1976), with few exceptions where colour can aid performance in such tasks (see Bramao, Reis, Petersson, & Faísca, 2011, for a review). In their majority, theories of object recognition predict and account for data showing shape as the primary feature by which objects are perceived and represented in memory (e.g., Biederman, 1987; Marr, 1982; Tarr & Bülthoff, 1998).

A more consistent picture has emerged from studies examining recognition memory for objects, using old-new recognition tasks to examine whether colour is an enduring property of object representations. In most old-new recognition tests, participants report during the test phase whether or not an object was seen in an earlier study phase. The majority of old-new recognition studies have examined the role of colour on recognition memory by comparing conditions where there is no change in colour between study and test against conditions where there is a change in colour. If the change in colour (e.g., from colour to achromatic or vice versa; or from one colour to another) influences recognition memory performance, then such a finding is interpreted as indicating that colour was encoded during study and the decrement in performance in that condition is due to the discrepancy between the memory representation, which contains colour, and the perceptual representation of the test image, which does not (e.g., Tulving & Thomson, 1973).

Old–new recognition studies have shown that colour aids retrieval from memory by providing an effective cue for retrieval of object representations (e.g., Cave et al., 1996; Nicholson & Humphrey, 2003; Vernon & Lloyd-Jones, 2003; Zimmer & Steiner, 2003) and representations of scenes of natural environments (e.g., Gegenfurtner & Rieger, 2000; Gegenfurtner et al., 1998; Wichmann et al., 2002). This advantage of colour as a retrieval cue generalises to objects that are not associated with a specific colour (e.g., Cave et al., 1996; Brady, Konkle, Alvarez, & Oliva, 2013; Hanna & Remington, 1996; Utochkin & Brady, 2019), and regardless of whether attention has been drawn to the colour of objects during the study phase (e.g., Brady et al., 2013; Cave et al., 1996). Furthermore, the colour and shape of images of simple forms (e.g., Fougnie & Alvarez, 2011; Hanna & Remington, 1996; Stefurak & Boynton, 1986) and everyday objects (e.g., Brady et al., 2013; Utochkin & Brady, 2019) have been shown to be stored independently, and independently influence memory retrieval. For instance, Brady et al. (2013; Exp.1) had observers study familiar coloured objects. They were then tested in a two-alternative forced choice task, either immediately after the study (short-delay condition) or after 3 days (long-delay condition). At test, observers chose which of two objects had been studied. In the *colour* condition, the target object (e.g., a pink sofa) would appear next to an identical object in a different colour (e.g., a green sofa). In the *state* condition, a target object (e.g., an open yellow deck chair) would appear next to the same object but in a different state (e.g., a closed yellow deck chair). At short delays, accuracy for the colour and state conditions did not differ, but with longer study-test delays, accuracy was much worse in the colour compared to the state condition. The finding that object colour information was forgotten to a greater degree than object shape information suggested that the two object properties are stored independently in memory.

Therefore, at present, there is little doubt that shape is a primary object attribute guiding many varying types of object recognition feats. There is also little doubt that colour is a represented feature in memory for objects and for natural scenes, and that under certain circumstances it influences object recognition performance. Yet, positive evidence for the representation of colour in episodic object memory is often complicated by the possibility that *encoding specificity* may be responsible for such observations (e.g., Tulving & Thompson, 1973). According to the encoding specificity principle, recognition memory performance would be superior when the recognition cues available for retrieval of an object (e.g., a specific shape, colour or word) were also present during the encoding of the same item. In other words, the greater the similarity in contexts between encoding and retrieval, the greater will be the success of retrieving the original item. Therefore, if a retrieval context includes a cue that was not part of the original encoding content (e.g., a new colour or texture), it will be overall weaker than a retrieval context that includes all the original cues.

Even with efforts to exclude encoding specificity as a plausible explanation for the detrimental effects of colour change between study and test (Brady et al., 2013; Nicholson & Humphrey, 2003) where object colour and shape information have been automatically encoded (i.e., no explicit instruction to attend to either object feature), the results do not allow conclusions regarding the *relative contribution* of shape and colour information in memory. That is because colour and shape were manipulated on different objects and not on the same object. Thus, a yet unanswered question remains as to how colour information fares relative to shape when retrieving an object from memory. So, when we study an object and store it in memory for a later test, is shape more likely to be the primary retrieval cue, or would colour be an equally viable retrieval cue? And how would one go about examining the *relative* importance of object shape and colour as retrieval cues from memory?

The question of the independent *and* relative contribution of the representations of different features in memory retrieval requires a paradigm where the features of the same object have the same chance of being retrieved at the same time, and where the effects of such retrieval attempts can be behaviourally measured. The *retrieval practice* paradigm (e.g., Anderson, Bjork, & Bjork, 1994; for recent reviews see Storm & Levy, 2012, and Murayama, Miyatsu, Buchli, & Storm, 2014), offers a powerful method for examining the features that are used during memory retrieval because it offers an indirect measure, where evidence that the feature or features in question are encoded and used for retrieval is a by-product of normal processing rather than explicitly examined.

The retrieval practice paradigm has been previously used to implicitly probe interference *between* feature-based representations in other types of memory (e.g., Reppa, Worth, Greville, & Saunders, 2013; Tempel & Frings, 2013, 2014a, 2014b), and *within* feature-based representations (e.g., retrieval of a feature can impair memory of other related features of the same object; Fan & Turk-Browne, 2013). As outlined below, retrieval practice provides a means to *indirectly* probe the *relative contribution* of different object attributes to memory performance.

## Using retrieval practice to probe the relative contribution of retrieval cues

A typical retrieval practice paradigm involves giving participants a list of category-exemplar word pairs to study (e.g., Fruit–Orange, Fruit–Banana, Body–Leg). Subsequently, in a retrieval practice session, participants are asked to retrieve half of the target exemplars from half of the categories (e.g., Fruit–Or___). Practiced categories are labeled ‘Rp’. Items from these practised categories that are individually practised are labeled ‘Rp+’ (e.g., Fruit–Orange), while non-practised items from the same practised categories are labeled ‘Rp–’ (e.g., Fruit–Banana), and non-practised items from non-practised categories are labeled ‘Nrp’ (e.g., Body–Leg). Following the practice phase, participants must recall all studied exemplars. Typically, recall is facilitated for practised (Rp+) items, but impaired for items that were not practised (Nrp and Rp–). Critically, memory for the unpractised exemplars from the practised category (Rp– items, e.g., Fruit–Banana) is poorer than memory for unpractised items whose category did not appear during the retrieval practice phase (Nrp items, e.g., Body–Leg). Anderson et al. (1994) termed this pattern of impaired recall *retrieval-induced forgetting* or *RIF*.

RIF is the observable measure of competition during practice in memory amongst the target (Rp+) and non-target (Rp–) item representations on the basis of shared properties between the two (e.g., Anderson, Green, & McCulloch, 2000b). Examining the properties of objects that guide competition effects in memory, and thus yield significant RIF, we can make inferences about the contents of an item’s representation. In the above example, significant RIF for Rp– items (e.g., Banana) suggests that the *explicitly* defined *category* (Fruit) is represented in the Rp+ (e.g., Orange) and Rp– item representations. Categories that are *implicitly* defined have also been known to elicit significant RIF (e.g., Ciranni & Shimamura, 1999; Reppa et al., 2013; Tempel & Frings, 2013, 2014a, 2014b).

Both colour and shape can act as *categories* that independently drive competition effects in memory.^1^ In the first and only study of this kind, Ciranni and Shimamura (1999) showed that retrieval based on shape alone or on colour alone led to significant forgetting for other geometric shapes that shared the same property. In one study examining colour as a retrieval cue, their participants studied a set of 12 uniquely shaped stimuli grouped by colour (e.g., four green shapes, four orange shapes, and four purple shapes), and subsequently practised a subset of stimuli from two of the colour categories (e.g., two green shapes and two orange shapes). At test, unpractised shapes that shared the same colour as the practised items (i.e., the other two green and two orange shapes) were susceptible to significant RIF. This suggested that colour can act as an implicit category, leading to competition of items that share it in memory.

However, because the study by Ciranni and Shimamura was not designed to address the relative contribution of shape and colour information in driving competition effects in memory, shape and colour were never varied simultaneously as properties of the same object, or as part of the same experimental episode. This is an important question because when both shape and colour information is available to index an object in memory, as in the case of complex objects, there is a possibility that during retrieval, shape primarily drives competition effects and colour can play only a negligible (e.g., Nicholson & Humphrey, 2003) or a short-lasting role (e.g., Brady et al., 2013). In the current studies, discovering the relative magnitude of competition of the two properties during practice would inform us on the *relative contribution* of colour and shape as retrieval cues for complex objects.

### Overview of experiments

Two experiments examined the relative contribution of colour and shape to object memory retrieval. As visual objects do not easily lend themselves to memory *recall* tasks, which are widely used with word stimuli, the current experiments utilised a *recognition practice* paradigm (e.g., Maxcey, 2016; Maxcey, McCann, & Stallkamp, 2020; Maxcey, Glenn, & Stansberry, 2018; Maxcey & Woodman, 2014; Reppa, Williams, Worth, Greville, & Saunders, 2017), where old–new recognition is performed during both the practice and the test phases of the retrieval practice paradigm. The overarching rationale was that if visual properties, such as shape and colour, drive object memory retrieval, then unpractised objects sharing either of those properties with the practised objects would compete during practice, would create interference, and, consequently be susceptible to RIF.

Recognition practice with a subset of studied objects (as opposed to the typical cued-recall practice described earlier) has been shown to successfully induce significant recognition-induced forgetting^2^ for unpractised (Rp–) objects (e.g., Maxcey & Woodman, 2014; Reppa et al., 2017). Note that although we used an old–new recognition task in both the practice and test phases in the current studies, it is the act of retrieval of information from memory during the practice phase that is critical in the paradigm, irrespective of whether it was cued recall or recognition. Indeed, the retrieval process mediating the recollection component of recognition tasks has been shown to mediate recall tasks (e.g., Brown, 1976; Mandler, 1980).

**Fig. 1:**
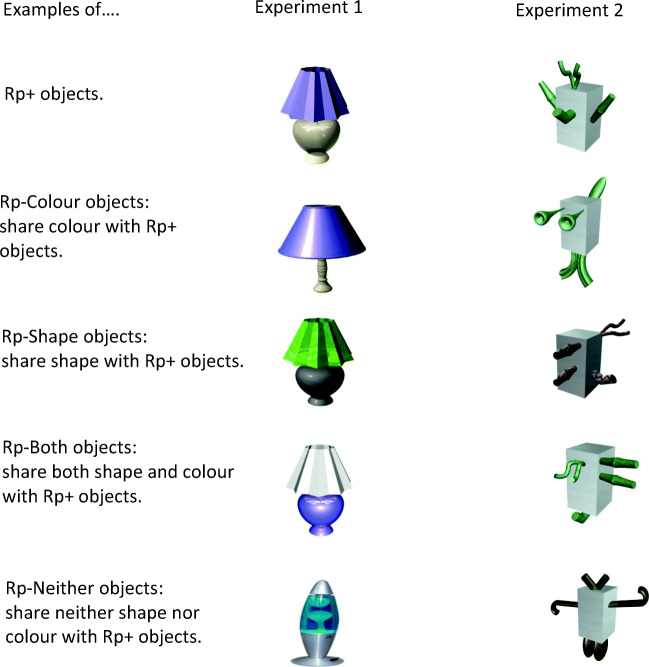
Examples of objects in the Rp conditions in the current experiments. The design is illustrated for familiar (Experiment 1) and novel objects (Experiment 2). See text for details

Participants in the practice group were exposed to familiar or novel objects and asked to memorize them. Then, during the practice phase, participants were shown a subset of the studied objects with which they performed an old–new recognition task. For each practised (Rp+) object there were four types of matched unpractised (Rp–) objects (see Fig. 1 for examples). Two types of Rp– objects were the critical conditions: Rp– objects that shared the *same shape* but had *different colour* to the practised objects (Rp–Shape), and Rp– objects that shared the *same colour* but had *different shape* to the practised objects (Rp–Colour). For completeness, we included Rp– objects that shared *neither shape nor colour* with the practised objects (Rp–Neither), and objects that shared *both shape and colour* with the practised objects (Rp–Both), but the colour assignment to the object parts was reversed (see *Apparatus and materials* for details). In the *test phase* participants performed an old–new recognition task on all the studied objects.

## Experiment 1

In Experiment 1, participants were exposed to familiar everyday objects (e.g., tables, chairs) before engaging in a recognition practice phase whereby they performed old–new recognition on a subset of the studied objects. The key question was whether recognition practice of an object impairs memory for unpractised objects that share only the same shape (Rp–Shape), or only the same colour (Rp–Colour) with the practised (Rp+) objects. If both shape and colour are represented in object memory, then both properties should drive competition effects in memory, and thus RIF would be present both for unpractised objects sharing only shape (Rp–Shape) and for those sharing only colour (Rp–Colour) with the practised objects. The relative magnitude of RIF in the Rp–Shape and Rp–Colour conditions would further indicate the strength or contribution of these two types of property at retrieval.

Sharing *both* shape and colour features with the practised object (e.g., Rp–Both) was also expected to produce significant RIF, possibly of similar magnitude as Rp–Colour and Rp–Shape objects (see Reppa et al., 2013). Finally, RIF was expected for Rp–Neither objects as they shared category (e.g., chairs) but neither colour nor shape with the practised objects, which has been previously shown to lead to significant RIF for visual objects (e.g., Maxcey & Woodman, 2014; Reppa et al., 2017). However, it seemed reasonable to expect that RIF magnitude might be lower for Rp–Neither objects compared to objects sharing both category and visual features (i.e., Rp–Colour, Rp–Shape, and Rp–Both).

### Method

#### Participants

Forty Swansea university students over the age of 18 years took part in the study in exchange for course credit. One group of 20 participants were allocated to the recognition-practice group (seven males and 13 females), and a different group of 20 participants (four males, 16 females) were allocated to the control group (no recognition practice). All reported normal or corrected-to-normal vision and normal colour vision. All were native English speakers and naïve to the purpose of the experiment.

#### Apparatus and stimuli

Experiment 1 was run on a Dell OptiPlex GX520 computer connected to a 15.5-in. LCD monitor. Stimulus presentation, trial randomisation, and recording of responses and response times were controlled via E-prime (version 2.0).

Object images were taken from Art Explosion 750,000 and the World Wide Web. The images were modified using Strata 3D pro and Adobe Photoshop and fit within a square (not visible during presentation) of approximately 10 × 10 cm with a resolution of 71 dpi. When viewed from a distance of 60 cm, the objects did not exceed 9.52 × 9.52° of visual angle.

**Fig. 2:**
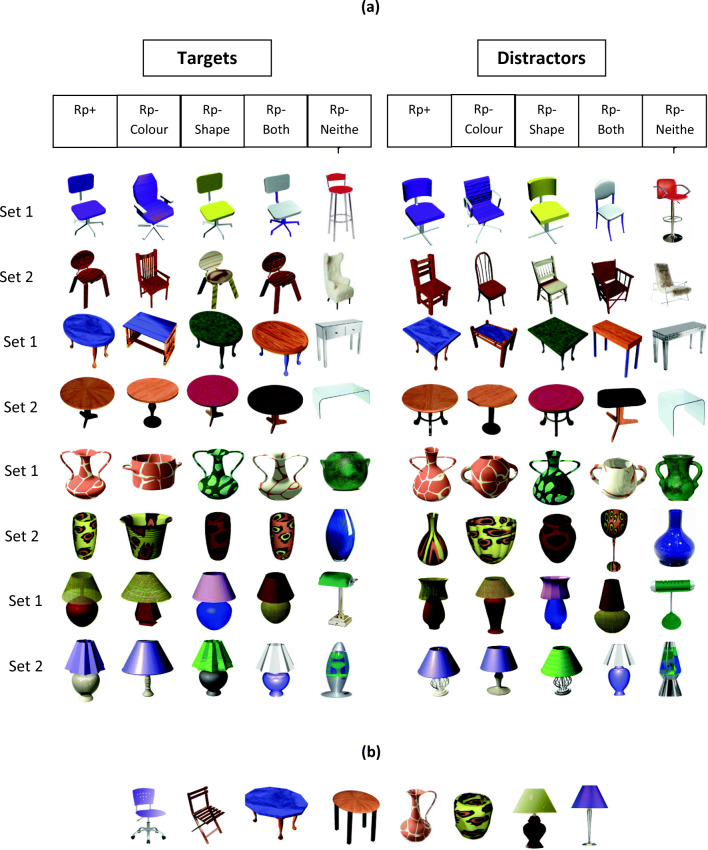
(**A**) Target and distractor objects used as stimuli in Experiment 1. (**B**) The distractor objects, one for each practised object, used during the recognition practice phases only

Figure 2 shows the 88 object images used in Experiment 1 (40 targets, eight distractors used in the practice phase only, and 40 distractors used in the test phase only). The stimuli were pictures of everyday objects belonging to one of four different categories: Tables, Chairs, Lamps and Vases – although these names were never mentioned to the participants.

In each object category (e.g., tables) there were two object sets (set 1 and set 2; Fig. 2, Panel A, five leftmost columns). For each object set there was a single Rp+ object for all participants in the recognition practice group. Corresponding Rp–Shape objects shared the exact same shape with Rp+ objects but differed in colours. Rp–Colour objects differed in shape from the Rp+ objects but had the same colours and texture as them. Rp–Both objects shared exactly the same shape and the same colours as the Rp+ objects, but the colours of the parts were re-assigned. For instance, if the Rp+ object was composed of a dark blue table top and light brown legs, then the Rp–Both object would be identical in shape to the Rp+ object, but the table top would be light brown and the legs would be dark blue. Finally, the Rp–Neither objects shared neither shape nor colour with Rp+ objects.

Once all the target (old) objects were created, distractor (new) objects (Fig. 2, Panel A, five rightmost columns) were made for each target object. Distractor objects were identical to targets in terms of colour (and colour combinations), as well as being similar to the targets in terms of overall shape configuration, and different from the targets in the shape of their individual parts. For the Rp+ objects, two distractor objects were created using the aforementioned constraints; one distractor object that was used as a distractor during the test phase only (Fig. 2, Panel A), and another that was used during the recognition practice phases (Fig. 2, Panel B).

#### Design

For the practice group, a repeated-measures design was used manipulating Practice with two levels: practised versus unpractised categories, and Item Type with five levels: Rp+ (practised objects), Rp–Shape (objects sharing shape with Rp+ objects), Rp–Colour (objects sharing colour with Rp+ objects), Rp–Both (objects that shared both the same shape and the same colour with the Rp+ objects), Rp–Neither (objects that did not share shape or colour with Rp+ objects).

For the control group a repeated-measures design was used manipulating only item type with five levels: Rp+, Rp–Colour, Rp–Shape, Rp–Both and Rp–Neither. As there was no recognition practice for the control group, those terms were irrelevant. However, control participant performance served as a baseline for the assessment of RIF. Furthermore, control group performance on each item type would help ensure that any effects of Item Type (which were of key interest here) were not caused by effects of baseline discriminability of each item type in memory. The dependent variable for both groups was target discriminability, expressed in terms of *A’* (Snodgrass, Levy-Berger, & Haydon, 1985).

#### Procedure

Participants were seated individually in a quiet room approximately 60 cm from the computer monitor. Participants in the recognition practice group completed a single study phase, three recognition practice phases separated by filler tasks, and a test phase. Control participants completed the study phase, a filler task and the test phase, but not the recognition practice phase. The filler task took as long to complete as the recognition practice phases did for the practice group participants.

##### Study phase

There was a single study phase, the same for both groups, where the 40 target objects appeared one at a time at screen centre, in a random order. Participants studied each object for 5 s and were told to expect a later memory test of the objects shown during the study phase.

##### Recognition practice phases

Only the practice group participants completed the recognition practice phase. Consistent with previous recognition practice experiments, participants completed three practice phases (e.g., Maxcey & Woodman, 2014; Reppa et al., 2017). In each of the practice phases, four of the studied objects were practised (two objects from two different object categories, e.g. two tables and two chairs) together with four distractor objects – one distractor object for each Rp+ object. Therefore, in each practice phase eight objects appeared (one at a time): four Rp+ objects and four distractor objects, yielding a total of 24 practice trials per participant across the three practice phases. In each practice phase, objects appeared at screen centre individually, in random order, with phases separated by filler tasks. During recognition practice, participants indicated whether they believed each object had been studied or not in the earlier phase (the study phase), by pressing either the Q or P key on a QWERTY keyboard. Half of the participants responded ‘Yes’ by pressing P with their right hand and ‘No’ by pressing Q with their left hand, with the response key reversed for the other half of the participants. There were equal numbers of old and new stimuli, and thus equal numbers of correct ‘Yes’ and ‘No’ answers. The practised categories for half of the participants (e.g., tables and chairs) were the unpractised categories for the other half of participants (e.g., lamps and vases), and vice versa. Correct responses were followed by a ‘Correct’ message on the screen for 1 s, and incorrect responses were followed by an ‘Incorrect’ message on the screen for 1 s concurrently with a 500-Hz beep sound. Incorrect trials were not replaced. There was no time limit for responding.

##### Filler tasks

For the Practice group participants, filler tasks were used in-between the three practice phases and in-between the last practice phase and the test phase. The first and second filler tasks (between the first and the second practice phase, and between the second and third practice phase, respectively) lasted for 2 min and the third (between the third practice phase and the test phase) lasted for 5 min. Filler tasks required participants to list as many words as they could for each letter of the alphabet, for a range of categories (e.g., girls’ names, animals, capital cities) with a different category used after each practice phase. The Control group participants completed the same practice tasks, but without the recognition practice element.

##### Test phase

The two participant groups completed the same test phase. All objects that were presented in the study phase, as well as all their associated distractors (see Fig. 2, Panel A) were presented individually and in a random order at screen centre. The task was identical to that of the recognition practice phase with participants indicating whether they had seen each object during the study phase or not using the same response keys as recognition practice group participants did during the practice phase. Fast and accurate responding was emphasised, and corrective feedback was provided in the same way as during the practice phases.

### Results

#### Recognition practice success

Recognition practice success was measured in terms of high discriminability of the practised (Rp+) studied objects against the distractors used during practice. Recognition practice in Experiment 1 was successful, with target objects being successfully discriminated from distractor objects (Hits: *M*=.92, *SD*=.09; False alarms: *M*=.11, *SD*=.15, *A’*: *M*=.94, *SD*=.08).

#### Test phase analyses

**Fig. 3:**
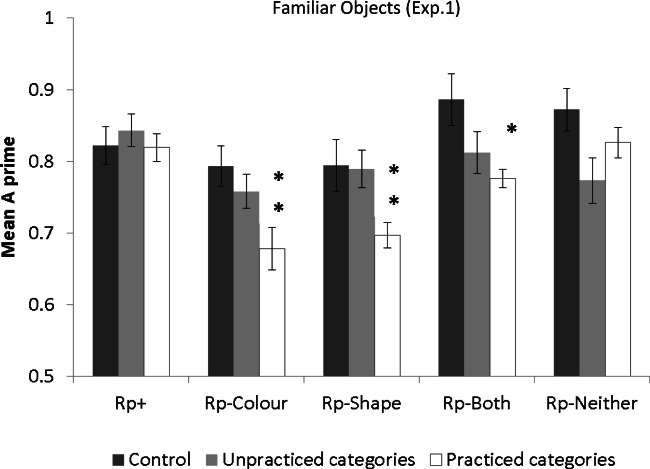
Mean A’ for each of the Rp conditions in Experiment 1 in the control group (dark grey bars) and the experimental group (light grey and white bars). Double asterisks denote significant difference between the Rp condition both from the equivalent condition in the control group and from the equivalent condition in the unpractised categories. The single asterisk for the Rp–Both RP condition denotes significant difference compared to the equivalent condition in the control group only. Error bars indicate standard error of the mean

Mean accuracy measures in Experiment 1 are shown in Table 1. The mean *A’* per Rp condition in Experiment 1 is shown in Fig. 3. The comparisons between Nrp and each of the Rp conditions were planned comparisons, based on the predictions outlined in the introduction. The Bonferroni correction was applied to all planned comparisons, and Cohen’s *d* effect sizes are reported for each.

**Table 1:** Mean proportion of hits (standard deviations in parenthesis), false alarms and *A’* scores per item type for Experiment 1, shown separately for the practice and the control group

		RP+	Rp–Colour	Rp–Shape	Rp– Both	Rp–Neither
Categories	Recognition practice group					
		Mean (*SD*)	Mean (*SD*)	Mean (*SD*)	Mean (*SD*)	Mean (*SD*)
Practised	Hits	.86 (.*08*)	.54 (.*19*)	.57 (.*23*)	.66 (.*22*)	.75 (.*18*)
	False Alarms	.38 (.*21*)	.32 (.*21*)	.29 (.*21*)	.24 (.*18*)	.24 (.*18*)
	*A’*	.83 (.*12*)	.68 (.*12*)	.72 (.*16*)	.79 (.16*)*	.84 (.*13*)
Unpractised	Hits	.81 (.*19*)	.64 (.*13*)	.68 (.*17*)	.74 (.*15*)	.64 (.*18*)
	False alarms	.27 (.*15*)	.28 (.*17*)	.25 (.*13*)	.26 (.*19*)	.23 (.*15*)
	*A’*	.85 (.*10*)	.76 (.*11*)	.80 (.*12*)	.82 (.*13*)	.79 (.*14*)
	Control group					
		Mean (*SD*)	Mean (*SD*)	Mean (*SD*)	Mean (*SD*)	Mean (*SD*)
	Hits	.84 (.*09*)	.68 (.*13*)	.63 (.*16*)	.81 (.*11*)	.80 (.*17*)
	False alarms	.37 (.*16*)	.24 (.*18*)	.22 (.*13*)	.18 (.*12*)	.19 (.*12*)
	*A’*	.83 (.*08*)	.81 (.*13*)	.79 (.*08*)	.89 (.*06*)	.88 (.*09*)

*Control group analysis*

A one-way repeated-measures ANOVA examining the effect of Item Type (Rp+, Rp–Colour, Rp–Shape, Rp–Both, and Rp–Neither) on *A’* scores showed a significant main effect, *F*(4, 19)=11.97, *p*=.003, *η*_*p*_^*2*^ =.39. Simple effects analysis to examine the main effect of Item type showed that Rp–Both yielded higher *A’* scores than Rp+, (*p*=.04) Rp–Colour (*p*=.02) and Rp–Shape items (*p*<.001). There were no other significant differences.

#### Practice group analysis

To estimate within-participants RIF and facilitation, a 2 (Practice: practised vs. unpractised categories) × 5 (Item Type: Rp+, Rp–Colour, Rp–Shape, Rp–Both, and Rp–Neither) repeated-measures ANOVA was first carried out on *A’* scores from the practice group. There was a significant main effect of Practice, *F*(1, 19)=4.46, *p*=.05, *η*_*p*_^*2*^ =.19, with higher *A’* scores for the unpractised compared to the practised object categories. There was also a significant main effect of Item Type, *F*(4, 76)=5.43, *p*<.001, *η*_*p*_^*2*^=.22. The interaction was only marginally significant, *F*(4, 76)=2.20, *p*=.07, *η*_*p*_^*2*^ =.10. Planned comparisons against the Nrp baseline showed significant RIF for Rp–Shape, *t*(19)=2.74, *p*=.02, *d*=.56 and Rp–Colour objects, *t*(19)=2.24, *p*=.03, *d*=69. There was no significant RIF for Rp–Both objects, *t*(19)=.78, *p*=.43, or for Rp–Neither objects, *t*(19)=.58, *p*=.57. Comparison between Rp+ and Nrp A’ scores, did not show significant within-participant facilitation, *t*(19)=.70, *p*=.49.

Next, between-participants RIF and facilitation was examined in a 5 (Item Type: Rp+, Rp–Colour, Rp–Shape, Rp–Both, and Rp–Neither) × 2 (Group: control vs. practice) mixed ANOVA with repeated measures on Item Type. The main effect of Item Type was significant, *F*(4, 152)=9.26, *p*<.001, *η*_*p*_^*2*^ =.20, as was the main effect of Group, *F*(1, 38)=11.26, *p*=.002, *η*_*p*_^*2*^ =.23. The interaction was marginally significant, *F*(4, 152)=2.01, *p*=.09, *η*_*p*_^*2*^ =.05. Planned comparisons to examine between-participant RIF showed a significant difference between the practice and the control groups in *A’* of Rp–Colour, Rp–Shape and Rp–Both objects [*t*(38)=2.80, *p*=.008, *d*=1.04; *t*(38)=2.41, *d*=.55, *p*=.02; and *t*(38)=2.91, *p*=.006, *d*=.21, respectively], but no significant RIF for Rp–Neither objects, *t*(38)=1.26, *p*=.21. There was no significant difference in *A’* between the practice and control group for Rp+ items, *t*(38)=.09, *p*=.92, suggesting no significant between-participant facilitation.

### Discussion

Experiment 1 showed significant RIF for Rp–Shape and Rp–Colour objects, suggesting that these two object properties are encoded and independently drive competition effects in memory. Conclusions about independence are warranted here because Rp–Shape objects share *only shape*, but not colour, with the Rp+ objects and, thus, recognition-induced forgetting can only occur due to Rp+ and Rp– items having shape in common. Similarly, Rp–Colour objects share *only colour*, but not shape, and, therefore, forgetting in the Rp–Colour condition can only be due to Rp+ and Rp– items having colour in common.

The lack of facilitation for Rp+ objects is not without precedence (for review, see Storm & Levy, 2012). In retrieval practice tasks, forgetting of unpractised items (Rp– items) has often been obtained even when retrieval attempts fail completely (e.g., Storm, Bjork, Bjork, & Nestojko, 2006) and in the absence of facilitation for practised items (e.g., Gómez-Ariza, Fernandez & Bajo, 2012; Gómez-Ariza, Lechuga, Pelegrina, & Bajo, 2005; Maxcey & Bostic, 2015; Maxcey, Bostic, & Maldonado, 2016). Thus, the lack of facilitation for Rp+ objects in Experiment 1 does not detract from the current observation of recognition-induced forgetting for unpractised objects.

Based on prior findings, we initially expected that we might find significant RIF for Rp–Neither objects because they shared the same category as the practised objects (e.g., Maxcey & Woodman, 2014). However, there was no evidence of RIF for these objects, which may be due to their visual distinctiveness from the large number of similar objects in their category (i.e., they differed on multiple dimensions, such as shape, colour, texture and material; e.g., see the Rp–Neither example objects in Fig. 1b). The distinctiveness of the Rp–Neither objects may have encouraged participants to become aware of the differences among the exemplars *within* different categories and may have protected them from forgetting (e.g., Macrae & Roseveare, 2002; Smith & Hunt, 2000).

There was no within-participant RIF for Rp–Both objects. At first blush this lack of forgetting for Rp–Both objects might seem to question the sensitivity of the paradigm to detect the object features that it is supposed to measure. Does the paradigm fail to detect a feature (e.g., colour) when it is presented together with another feature (e.g., shape)?

**Fig. 4:**
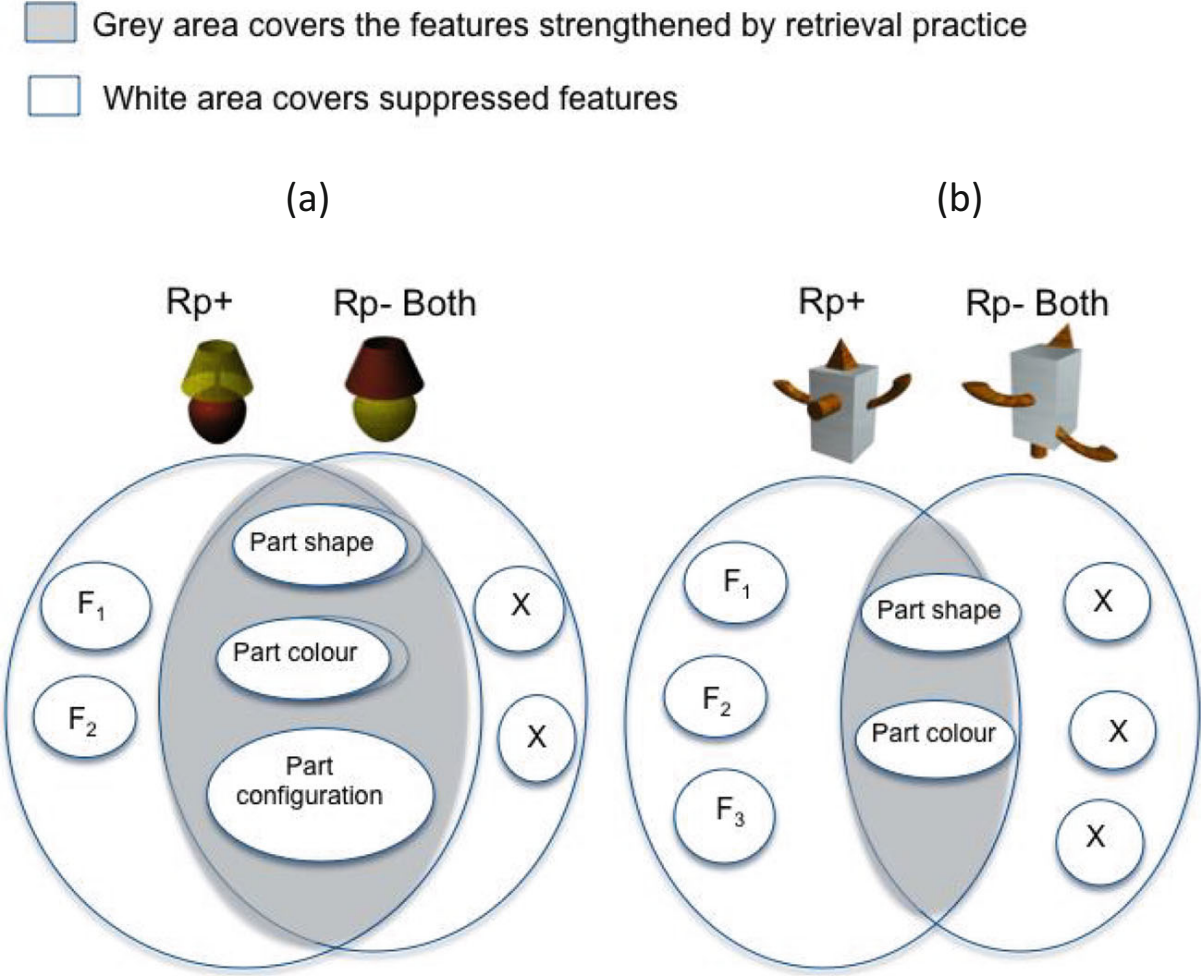
Illustration of target-competitor similarity (adapted from Anderson, 2003). Objects here (larger circles) are represented as sets of features (F1=feature 1, etc.). Here, three of those features are part shape, part colour and part configuration. Similar objects, like the two lamps on the left, overlap in feature space (as represented by overlapping larger circles). Retrieval or recognition practice is assumed to increase the activation of practised features (i.e., part shape, colour and configuration) and to inhibit other features of the competing similar object (represented as circles with Xs). When target–competitor similarity is high, as in the case of the familiar objects experiments (Exp. 1, Panel A), then a greater proportion of the competitor’s (Rp- Both) features overlap with the practised item (Rp+) and are strengthened, masking or even eliminating any effects of suppression on the remaining features. The opposite is expected when target-competitor similarity is low, as in the case of the novel objects experiments (Exp. 2, Panel B). With low target-competitor similarity, fewer of the competitor’s features overlap with the target object, while the remaining non-overlapping features are susceptible to suppression, and yield significant RIF

The absence of RIF for Rp–Both objects may be explained by the model of distributed item representation in memory proposed by Anderson and colleagues (e.g., Anderson et al., 2000a, b; Anderson & Spellman, 1995). According to this model, the probability of an item being recollected is dependent on the overlap of features between target (i.e., Rp+) and competitor (i.e., Rp–) representations (see Fig. 2). With *high* feature overlap (e.g., Fig. 4, Panel A) the net activation of primed features in the competitor representation will result in the elimination of recognition-induced forgetting, or even facilitation for the competitor (in this case Rp–Both objects). Conversely, with *moderate* overlap (e.g., Fig. 4, Panel B) between target and competitor the net suppression of features in the competitor representation would be expected to outweigh the net facilitation from primed features shared with the Rp+ item (e.g., category), and thus yield significant recognition-induced forgetting. In Experiment 1, the feature overlap between Rp+ and Rp–Both objects can be considered high (i.e., the two object types shared object parts, part configuration, and part colour), which may be the reason for the lack of significant RIF. Therefore, for feature-based representations like the ones we are tapping into here (as evidenced by the presence of RIF for Rp–Colour and Rp–Shape objects), the lack of RIF in the Rp–Both condition may confirm the sensitivity of the paradigm to object features. This issue is revisited in Experiment 2.

## Experiment 2

Experiment 2 used novel objects in order to provide converging evidence for the findings of Experiment 1 and for previous studies (e.g., Brady et al., 2013) concerning the independent representation of colour and shape in object memory. Experiment 2 aimed to also provide converging evidence for the Experiment 1 findings regarding the relative contribution of shape and colour information to memory retrieval, and to improve on its approach in several ways. For familiar recognisable objects, verbal labels are often automatically elicited and are therefore connected with the visual information (e.g., Paivio, 1991). Such labels can help bind colour and shape information to the object’s identity, and so memory effects may be mediated by more than the representation of colour as a perceptual feature (e.g., Tanaka, Weiskopf, & Williams, 2001). Novel objects help eliminate the use of a verbal coding strategy (e.g., Nicholson & Humphrey, 2003), allowing a purer examination of the representation of colour in object memory as a perceptual feature.

Experiment 2 allowed a much stronger test for the capacity of colour information to cause competition effects in memory. In Experiment 1, colour was still relevant to the task, because some objects could only be differentiated from others on the basis of colour. For instance, Rp+ and Rp–Shape objects were identical apart from their colour. This was remedied in Experiment 2 because with novel objects we were able to alter aspects of shape in such a way so that all objects could be distinguished on the basis of their shape alone, rendering colour entirely task irrelevant.

**Fig. 5:**
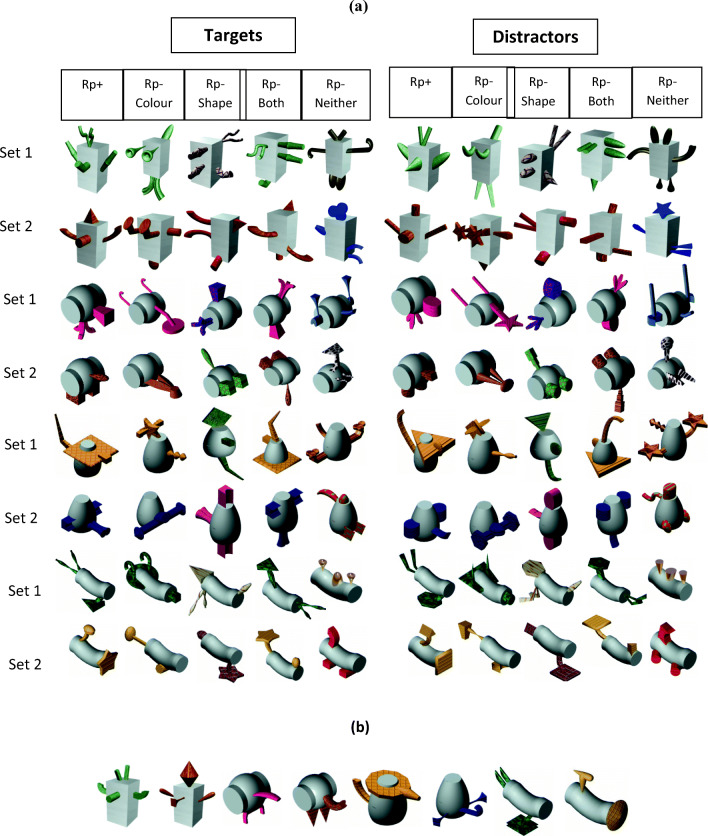
(**A**) Target and distractor objects used as stimuli in Experiment 2. (**B**) The distractor objects, one for each practised object, used during the recognition practice phases only. The central components of all objects within a category in Experiment 2 were identical, although they may appear slightly different here due to re-sizing

Finally, the use of novel objects in Experiment 2 allowed for unique spatial configurations across the different classes of stimuli, which would help address some potential concerns with the Rp–Both condition in Experiment 1. Specifically, in Experiment 1, Rp–Both and Rp+ objects shared the same colour, the same shape of individual parts, *and* the same spatial configuration of parts in order to make real, recognizable objects (see Figs. 1 and 2). This was unavoidable as creating familiar Rp–Both objects that did not share the same part spatial configuration with the Rp+ objects would have required scrambling their parts, effectively rendering them as non-objects. Based on the possibility that similarity between Rp–Both and Rp+ objects (i.e., high target-competitor similarity; Anderson et al., 2000a, b) may have been the reason for the lack of RIF for Rp–Both objects, similarity in Experiment 2 was reduced by having unique spatial configurations for Rp–Both items (Fig. 5) with the expectation of significant RIF under these stimulus conditions.

### Method

#### Participants

Forty Swansea University students took part in the experiment in exchange for participant pool credits. Twenty participants (eight males and 12 females) were allocated to the recognition practice group and 20 (four males and 16 females) to the control (no recognition practice) group. All participants reported normal or corrected-to-normal vision and normal colour vision.

#### Apparatus and stimuli

The apparatus used was exactly the same as in Experiment 1. The stimuli used were pictures of novel objects (adapted from Michael Tarr’s stimulus database found at: https://wiki.cnbc.cmu.edu/TarrLab). They were modified in Strata 3D Pro and Adobe Photoshop. The novel objects were created so they could be grouped, via the shape of the central components, into four different informally labelled categories: Ballerinas, Mowers, Probes and Tubes (Fig. 5). These were fictitious names for the benefit of the experimenters, and participants were naïve to these names. The central component of all objects in all categories was the same colour (i.e., grey). Each object image fitted within a (non-visible during the experiment) 10 × 10 cm square (355 × 420 pixels) with a resolution of 71 dpi.

Eighty-eight objects were used in total: 40 targets, eight distractors for the practice phase only, and 40 distractors used in the test phase only. The basic principles of creating the Rp–Shape, Rp–Colour, Rp–Both, and Rp–Neither objects were similar to Experiment 1. The Rp–Shape objects differed in colour, but shared the same parts as Rp+ objects, but unlike Experiment 1, the parts were arranged in a different spatial configuration from the parts in the Rp+ objects. The Rp–Colour objects shared the same colours as the Rp+ objects but differed in both part shape and part configuration. The Rp–Both objects were created by changing the configuration of the parts of the Rp+ objects (Fig. 2a), ensuring that the Rp–Both objects shared the same parts and colour as Rp+ objects but the parts were in a different spatial configuration. Therefore, Rp–Shape and Rp–Both objects had the same object parts as Rp+ objects, but the spatial arrangement of the parts differed in each of the three conditions outlined. Finally, the Rp–Neither objects differed from the Rp+ objects both in terms of colour, part shape and part configuration.

#### Design and procedure

Experiment 2 employed the same design as Experiment 1. The procedure was identical to Experiment 1, with the exception that there were two consecutive study phases (as opposed to only one as was the case in Experiment 1), where participants studied the objects twice, once in each study phase, with a self-paced break in-between the two phases. Pilot work indicated that participants required two study phases in order to learn the objects to a comparable degree of accuracy as the familiar objects of Experiment 1. Data analysis procedure was the same as in Experiment 1.

### Results

#### Recognition practice success

During recognition practice, participants successfully discriminated target from distractor objects (Hits: *M*=.89, *SD*=.13; False alarms: *M*=.18, *SD*=.15, A’: *M*=.92, *SD*=.10).

#### Test phase analyses

**Fig. 6:**
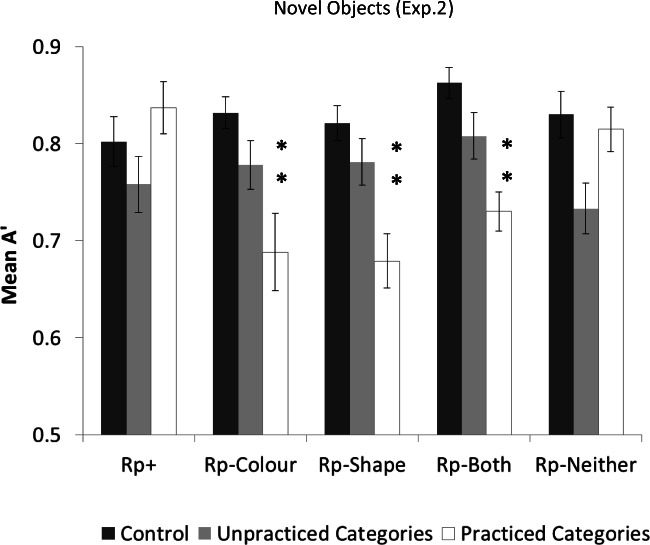
Mean A’ for each of the Rp conditions in Experiment 2 in the control group (dark grey bars) and the experimental group (light grey and white bars). Double asterisks denote significant difference between the Rp condition both from the equivalent condition in the control group and from the equivalent condition in the unpractised categories. Error bars indicate standard error of the mean

Mean *A’* scores per condition, as well as hits and false alarms in Experiment 2, appear in Table 2. Figure 6 shows the mean *A’* per Rp condition per group.

**Table 2:** Mean proportion of hits (standard deviations in parenthesis), false alarms and A’ scores per item type for Experiment 2, shown separately for the practice and the control groups

		RP+	Rp–Colour	Rp–Shape	Rp– Both	Rp–Neither
Categories	Recognition practice group					
		Mean (*SD*)	Mean (*SD*)	Mean (*SD*)	Mean (*SD*)	Mean (*SD*)
Practiced	Hits	.85 (.*09*)	.51 (.*25*)	.44 (.*21*)	.52 (.*19*)	.64 (.*15*)
	False alarms	.34 (.*20*)	.24 (.*15*)	.21 (.*19*)	.22 (.*14*)	.17 (.*13*)
	*A’*	.84 (.*12*)	.69 (.*18*)	.68 (.*17*)	.73 (.*13*)	.81 (.*10*)
Unpractised	Hits	.76 (.*16*)	.72 (.*16*)	.57 (.*23*)	.74 (.*17*)	.58 (.*15*)
	False alarms	.37 (.*19*)	.31 (.*25*)	.18 (.*10*)	.29 (.*21*)	.24 (.*16*)
	*A’*	.76 (.*17*)	.78 (.*16*)	.78 (.*11*)	.81 (.*11*)	.73 (.*16*)
	Control group					
		Mean (*SD*)	Mean (*SD*)	Mean (*SD*)	Mean (*SD*)	Mean (*SD*)
	Hits	.75 (.*16*)	.75 (.*16*)	.74 (.*14*)	.75 (.*16*)	.79 (.*15*)
	False Alarms	.30 (.*19*)	.26 (.*17*)	.26 (.*17*)	.16 (.*12*)	.28 (.*19*)
	*A’*	.80 (.*11*)	.83 (.*08*)	.82 (.*09*)	.86 (.*07*)	.83 (.*11*)

#### Control group analysis

A one-way repeated-measures ANOVA examining the effect of Item type (Rp+, Rp–Colour, Rp–Shape, Rp–Both, and Rp–Neither) on *A’* scores showed no significant main effect, *F*(4, 76)=1.60, *p*=.182, *η*_*p*_^*2*^ =.08.

#### Practice group analysis

To estimate within-participants RIF and facilitation as a result of practice, a 2 (Practice: practised vs. unpractised categories) × 5 (Item Type: Rp+, Rp–Colour, Rp–Shape, Rp –Both , and Rp–Neither) repeated-measures ANOVA was first carried out on *A’* scores from the practice group. Neither main effect was significant [Practice: *F*(1, 19)=1.18, *p*=.29, Item Type: *F*(4, 76)=1.92, *p*=.12], but the interaction was significant, *F* (4, 76) = 5.42, p<.001, *η*_*p*_^*2*^ =.22. Planned comparisons between practised and unpractised items for each item type showed significant RIF for Rp–Shape, *t*(19)=2.31, *p*=.03, *d*=.70, Rp–Colour , *t*(19)=2.67, *d*=.53, *p*=.01, and Rp–Both objects, *t*(19)=2.2, *p*=.04, *d*=.66. There was a non-significant trend for facilitation for Rp–Neither objects, *t*(19)=.58, *p*=.06. There was no significant difference for Rp+ objects between practised and unpractised categories, *t*(19)=1.61, *p*=.12, thus no significant within-participant facilitation.

Next, between-participants RIF and facilitation was examined in a 5 (Item Type: Rp+, Rp–Colour, Rp–Shape, Rp–Both, and Rp–Neither) × 2 (Group: control vs. practice) mixed ANOVA with repeated measures on Item Type. The main effect of Item Type was significant, *F*(4, 152)=3.88, *p*=.005, *η*_*p*_^*2*^ =.09, as was the main effect of Group, *F*(1, 38)=12.51, *p*<.001, *η*_*p*_^*2*^ =.25. The interaction was also significant, *F*(4, 152)=6.12, *p*<.001, *η*_*p*_^*2*^ =.14. Planned comparisons showed that control group participants yielded higher A’ than the practice group participants for Rp–Colour, Rp–Shape and Rp–Both objects [*t*(38)=3.36, *p*=.002, *d*=1.03, *t*(38)=3.41, *p*=.002, *d*=.93, and *t*(38)=4.06, *p*<.001, *d*=1.24, respectively], but there was no difference for Rp–Neither objects, *t*(38)=.43, *p*=.66. There was no significant difference in *A’* between the practice and control group participants for Rp+ objects, *t*(38)=.94, *p*=.35, suggesting no significant between-participant facilitation.

### Discussion

The use of novel objects in Experiment 2 produced a strikingly similar outcome to Experiment 1 despite the drastic differences in object design. The findings of significant RIF for Rp–Colour objects confirmed previous findings that colour information is a pervasively represented object property – a finding that persists for novel objects with no prior semantic associations between shape and colour, as well as for objects that lack colour or shape diagnosticity.

Experiment 2 provides even stronger evidence for the role of colour as a retrieval cue for visual objects. That is, in Experiment 1, there were some instances where target objects could only be distinguished from others based on colour (e.g., Rp+ and Rp–Shape), which may have made it hard or impossible to ignore colour. However, in Experiment 2 all objects were different in terms of overall shape (shape of the parts and/or their configuration, depending on condition), making colour unnecessary to distinguish among target objects. Even under these conditions, where shape was entirely diagnostic of object identity and would have been the only cue used for memory retrieval, colour was encoded and used for retrieval of objects from memory during practice – objects sharing either colour or shape, or both, with the practised objects independently competed for retrieval with the practised objects and were thus susceptible to significant RIF of similar magnitude.

Finally, a goal of Experiment 2 was to decrease similarity of Rp+ and Rp–Both objects in an effort to induce forgetting in the Rp–Both condition as predicted by the Anderson and colleagues’ account. The finding of significant RIF for Rp–Both objects in Experiment 2 supported this explanation, suggesting that discriminability was influenced by changes in similarity between practised and unpractised objects.

## General discussion

Colour information clearly plays a role in object recognition, both by aiding the encoding of the image of a visual object in memory and by acting as an efficient retrieval cue for both isolated objects and for objects embedded in natural scenes (e.g., Gegenfurtner & Rieger, 2000; Wichmann et al., 2002; Gegenfurtner et al., 1998), especially for objects whose colour is strongly associated with their identity, or when colour is a prominent (e.g., Hanna & Remington, 1996; Stefurak & Boynton, 1986; but see Cave et al., 1996, and Brady et al., 2013, for exceptions) or the only encoding cue (e.g., Brady et al., 2013; Ciranni & Shimamura, 1999).

But what is the contribution of colour *relative to* shape in memory performance? The current studies examined this question for objects where both shape and colour were prominent during encoding (Experiment 1) and where colour was less prominent than shape during encoding (in Experiment 2 all objects to be learned could be differentiated on the basis of shape alone). A powerful way to answer this question was to use a task where the contribution of different features to memory retrieval is indirectly induced and measured. The use of the retrieval practice task allowed for such *indirect* probing of visual object memory representations and their *relative contribution* as cues to memory retrieval. The rationale was if shape and colour similarly influence memory retrieval, then unpractised (competitor) objects sharing a represented feature with the practised (target) objects should compete during practice and be susceptible to RIF. Conversely, if competition does not ensue, such as when a feature does not influence retrieval, then interference would be unlikely, and RIF would be absent.

Significant RIF emerged for objects sharing shape only, for objects sharing colour only, and for objects sharing both features with the practised objects. This is a particularly important finding given the primacy of object shape in object learning early in development (e.g., Landau, Smith, & Jones, 1988; Ritter et al., 2017) as well as its central role in object identification and recognition. Here we provide converging evidence that colour is a represented object feature in memory for objects and independently of shape, and show for the first time that its contribution to memory retrieval is comparable to that of shape – visual objects are indexed in terms of their shape and in terms of their colour, and both cues customarily are involved in the retrieval of objects from long-term memory.

### Implications for our understanding of retrieval-induced forgetting

#### Retrieval-induced forgetting emerges as a by-product of processing

In the current work, retrieval-induced forgetting or RIF was used as a tool to indirectly probe object representations in memory. The rationale was that if a feature is encoded and represented in long-term memory, then Rp– items containing this feature will compete in memory for retrieval during the retrieval of the Rp+ item, and this competition will be revealed by significant RIF for those items. All of this occurs as a by-product of normal processing.

But how did competition arise in the current experiments? In the conventional RIF paradigm where category-exemplar pairs are used, e.g., FRUIT —banana, FRUIT — orange, any presumed response competition during retrieval of FRUIT — banana, potentially leads to RIF for unpractised exemplars, e.g., FRUIT — orange. In this example, the category cue is explicit, in the sense that it is explicitly linked to each item, as well as being explicitly presented during the study phase.

The current design departed from the typical procedure in that both during practice and during test exemplars (objects in this case) were not explicitly linked with a category cue. So, how did response competition arise in such a design? Although each object was practised as a unique entity (e.g., in Experiments 1 and 2: “Did you see this particular object?”), different object features – object shape and object colour – were shared between different objects (see also Anderson & Spellman, 1995; MacLeod, 2002). Therefore, any competition between objects during recognition practice of a specific object would arise from the similarity of the practised object to other objects that share either the same shape *or* the same colour. In other words, competition in the current study arose from sharing categories, which were formed after incidental learning of the objects (e.g., Anderson & Spellman, 1995).

#### RIF is sensitive to feature-based episodic representations

Two findings from the current experiments suggest that RIF was mediated by episodic representations. First, the finding of RIF using a recognition task with distractors that were in the same category as the targets suggests that RIF operated on the episodic representations of the studied objects. Second, the finding of RIF using novel objects with no previous associations or semantic meaning suggests that again RIF was operating on the episodic representations of the studied objects. Both findings add to the evidence for RIF for episodically encoded perceptual properties (e.g., Ciranni & Shimamura, 1999), and show that interference can occur across multiple feature dimensions with much richer and more complex stimuli.

Theories regarding the representations underlying RIF were put to the test using the Rp–Both conditions of the current experiments. In the Rp–Both condition – where unpractised objects shared both shape and colour with the practised objects – RIF was non-significant in Experiment 1, but significant in Experiment 2. If shape and colour independently contribute to RIF, why was RIF elusive when both properties were combined in Experiment 1?

One explanatory framework that accounts for the inconsistent observation of RIF in the current studies is the distributed memory model proposed by Anderson and colleagues (2000a, b), according to which items in memory are represented in terms of features. In the context of retrieval (and recognition) practice the degree of net activation or suppression of a representation in memory is dependent on the degree of overlap in shared features between the target and competing representation. More specifically, representations of competing objects that share *many features* (e.g., part shape, part configuration, and part colour) with the target (Rp+) objects would be more likely to be primed during practice of the target, leading to net activation of the memory. In contrast, representations of objects that share *only some features* (e.g., part shape, part colour, but *not* part configuration) with the target object are more likely to be suppressed in memory during practice. Confirming the predictions of Anderson and colleagues’ (2000a, b) model, significant RIF was observed when Rp+ and Rp–Both objects were *moderately* similar, as in Experiment 2, but not when the two object types were *highly* similar. Combined, the results across the two experiments reported here regarding the Rp–Both condition demonstrate the sensitivity of RIF to episodic feature-based representations in object memory.

### Relationship of the current work with other paradigms

Granted, there are other, simpler, paradigms to examine whether an object feature is part of an object’s long-term memory representation, and used to guide memory performance – some of which were reviewed earlier. For instance, by varying the study-test time in an old–new recognition paradigm, Brady et al. (2013) elegantly showed that shape and colour are independently stored and retrieved from memory. However, in that paradigm, when memory for colour was examined (“Did you see the pink or green sofa at study?”), colour was the only available retrieval cue for the test with the shape of the two alternatives being identical, and thus uninformative. Similarly, when object state was the examined feature (“Did you see the open or the closed yellow chair at study?”), state was the only available retrieval cue, while the colour of the alternative choices was identical, thus uninformative. As shape and colour were never available retrieval cues for the same object, that study neither was designed to nor did it address the issue of the relative contribution of shape and colour in indexing and retrieving an object from long-term memory.

The question of the relative contribution of the two is more complex than the question of independence, and we needed a paradigm where colour and shape would be equally available retrieval cues for the same object. As an interference paradigm, retrieval practice is one ideally suited for the task. Firstly, it allows the probing of the representations of object features while minimizing attention to the examined features. This is because competition during memory retrieval is not in the participant’s conscious control, but a by-product of normal processing. Specifically, objects stayed the same in the study, practice, and test phases and only access to their representation as a result of competition would change, depending on how important the shared feature is in guiding memory retrieval.

Second, retrieval practice allows the probing of the relative contribution of shape and colour for retrieval of objects from memory. If object colour information is used for retrieval, then significant RIF would be expected when objects share the same colour (Rp–Colour) as the practised objects. The same line of argument applies to objects sharing shape (Rp–Shape) with the practised objects. Conversely, if a feature of an object, such as colour or shape, is not a feature driving memory retrieval then that feature would be unlikely to drive competition and induce interference; thus RIF would be absent for unpractised objects sharing that feature with the practised objects. Given that interference effects in memory between the practised and unpractised objects are typically revealed in RIF of related unpractised items, the design allows the comparison of RIF induced by colour against RIF induced by shape. Therefore, one strength of using an interference paradigm, such as retrieval practice, to examine mental representations of objects is its ability to implicitly probe those representations, thus avoiding the influence of task demands and determine whether the features in question are encoded and used for retrieval is a by-product of normal processing.

Similarly, other studies have taken the approach of using an interference paradigm, particularly in false memory (e.g., Lyle et al., 2006), to study the role of object features in long-term memory. For instance, in a typical false-memory task, participants would either view line drawings of everyday objects (e.g., a magnifying glass) or be asked to imagine visually similar objects (e.g., a lollipop). Increasing the number of perceptually similar studied pictures increases false claims of having perceived a picture of the imagined item. Those memory errors are typically associated with the recollection of specific features that were originally linked to the perceived items (e.g., location or colour; Lyle, Bloise, & Johnson, 2006; Lyle & Johnson, 2006, 2007) and at test misattributed to the imagined items. By manipulating attributes of perceived objects – e.g., shape and colour – one can observe whether the perceived features will be re-allocated to the imagined object.

In one such study, Lyle and Johnson (2006) had participants either view or imagine an everyday object. In one condition, participants saw or imagined objects that were visually similar in terms of shape (e.g., magnifying glass and lollipop) or dissimilar (belt and feather). At a source-monitoring test, they were given verbal labels of the studied objects and asked if the object was seen or imagined. Imagined objects (e.g., lollipop) were above chance likely to be recognised as having been seen in the colour of the perceptually similar actually seen object (e.g., red magnifying glass). Shape and colour of perceived objects can be automatically attributed to imagined objects – i.e., a red *magnifying glass* is more likely to lead to false memory of a red *lollipop*, but the picture of a *belt* does not reliably predict whether *feather* will become a false memory. This suggests that given the shape information, colour can be automatically attributed to an imagined object. In such studies, interference between actual and false memories in this case has been driven by shape

The false-memory paradigm has not, so far, been used to examine the content of visual object memories, but it has the potential to do so at least to some extent. For instance, one should be able to show that colour can drive false-memory content by having participants study objects, e.g., red magnifying glass, or imagining a fire-engine – both are related by colour (red). Then, during test participants are shown verbal labels of the seen and imagined objects and tested on their source memory (seen or imagined). If colour is explicitly indexed in memory and drives false-memory content, then one might find more false memories (attributing imagined objects as having been seen) for objects that are related to the seen objects by colour.

Even so, the recognition-induced forgetting task is better suited to examine the content of purely visual object representations in long-term memory, as visual object images can be used at study and test. In contrast, at least at present, false-memory paradigms typically use verbal labels as retrieval cues at test, limiting the question regarding the nature of object representations to everyday objects – thus not able currently to avoid issues of semantic associations potentially contributing to memory performance. The capacity of RIF to indirectly gauge competition in memory situates it ideally as a sensitive instrument to detect which features are represented in object memory. The current studies’ use of complex, multi-featured objects with both shape and colour acting as potential categorising cues demonstrated that both shape and colour guide retrieval of complex objects from memory, as both led to significant RIF effects.

### Alternative account of the current findings

Although we have interpreted the current findings as showing that shape and colour information are explicitly and independently represented in long-term memory for objects, there is another possibility that needs to be addressed. That is, the significant RIF for objects sharing features with the practised objects may reflect the strengthening of the association between two features – say Shape 1 and Colour 1 (e.g., Rp+ chair in Set 1) during retrieval, and this association during retrieval subsequently blocks the memory for another non-retrieved association – i.e., between Shape 2 and Colour 1 (e.g., Rp-Colour chair in Set 1). This possibility is predicted by non-inhibitory accounts of RIF (e.g., associative blocking: J.R. Anderson, 1983; Butler, Williams, Zacks, & Maki, 2001) according to which RIF is due to the strong practised memories (i.e., Rp+ items) blocking or interfering with the retrieval of weaker non-practised memories (i.e., Rp− items). As retrieval strengthens the association between a retrieval practice cue and the practised item (e.g., Fruit – cherry), it simultaneously weakens the association between this cue and other related but non-practised memories (e.g., Fruit – kiwi). As a result, RIF will occur whenever a strong practised item blocks retrieval of weaker non-practised items, such as when an Rp+ item is strengthened through retrieval practice.

Two findings in the current studies, however, make it unlikely that such blocking can account for the pattern of results. First, RIF was observed despite a lack of significant facilitation effects for Rp+ items. This would suggest that RIF cannot solely be due to the fact that the strength of memory associated with the Rp+ items blocks or interferes with the memory of unpractised items, as RIF could occur even when the items proposed to cause the memory blocking failed to be remembered. Second, the blocking accounts of RIF might predict a more uniform pattern of suppression of unpractised items than observed here. Instead, the magnitude of RIF differed between the Rp− objects, in a manner consistent with whether the objects contained any of the features of the practised objects, and their degree of similarity. Both lines of evidence would suggest that RIF in the different Rp- conditions (Rp−Shape, Rp−Colour, Rp−Both) was not solely dependent on the strengthening on Rp+ items, in other words not solely dependent on repetition during retrieval, but most likely on competition during retrieval guided by independent object feature representations.

### Implications for visual object representations in long-term memory

The current work provides converging evidence that colour can be used to categorise and retrieve objects in long-term memory independently of shape, even when it is not the only available grouping feature (unlike Ciranni & Shimamura, 1999, & Brady et al., 2013), and without any explicit instruction to attend to this feature dimension (unlike Hanna & Remington, 1996, and Stefurak & Boynton, 1986). The claim of shape and colour independence is warranted in the current studies on the basis of the significant forgetting for Rp–Shape and Rp–Colour objects. As Rp–Shape objects shared *only shape* but not colour with the Rp+ objects, forgetting in this condition could only come from the sharing of shape. Similarly, because Rp–Colour objects shared *only colour* but not shape with Rp+ objects, forgetting in the Rp–Colour condition could only come from the sharing of colour.

More importantly, the current study has shown that a visual object’s shape and colour are equally likely to be used to index and retrieve images of visual objects from long-term memory, with no difference between them as gauged by their strength of interference during the act of retrieval. This is a non-trivial finding – it shows for the first time that colour – far from being a secondary feature, only available when shape information is not sufficient – is used as a strong memory retrieval cue, even when shape would be sufficient (as in Experiment 2).

The current work has provided evidence from converging operations regarding the structure of shape representations in long-term memory. Specifically, competition in memory arose not only at the level of overall object configuration, i.e., when Rp–Shape objects shared the same parts and part configuration with the Rp+ objects, as in Experiment 1 (familiar objects), but also at the level of individual object parts, i.e., when Rp–Shape objects shared the same parts as Rp+ objects but were arranged in a different spatial configuration, as in Experiment 2 (novel objects). The finding that the shape of the individual object parts was sufficient to cause interference effects suggests that the retrieval practice paradigm taps into structured object representations in memory, complementing similar conclusions from visual attention paradigms (e.g., Arguin & Saumier, 2004; Behrmann, Moscovitch, Peterson, & Suzuki, 2006). The current studies provide converging evidence showing that individual object parts are sufficient to cause interference effects in long-term memory.

## Footnotes

1 Recently, research has shown that retrieving features of an object can influence the memory of other features of the same object (e.g., Fan & Turk-Browne, 2013), thus showing that the *object* can act as an implicit category that drives competition in long-term memory. Here, we examined whether *object features* themselves can act as categories independently of the other features of the same object, by examining whether retrieval of an object feature can impair memory of other objects bearing that feature.

2 Although the acronym RIF refers to retrieval-induced forgetting, the term is used in the current work to also denote recognition-induced forgetting. Note that the acronym RIF has been used to describe the specific type of memory impairment described here, even in the absence of a retrieval task during practice (e.g., Raaijmakers & Jakab, 2012; Verde, 2013). Furthermore, the use of the acronym RIF allows the current work to be related to the wider literature of retrieval-induced forgetting.
